# Splenic Abscesses With Different Modalities of Management: A Case Series

**DOI:** 10.7759/cureus.89325

**Published:** 2025-08-04

**Authors:** Chakravarthi Vainatheya Peramur Hemambjam Nallan, Arulappan T, Sivaraja PK

**Affiliations:** 1 General Surgery, Sri Ramachandra Institute of Higher Education and Research, Chennai, IND

**Keywords:** computed tomography (ct), diabetes mellitus, image-guided drainage, splenectomy, splenic abscess

## Abstract

Splenic abscesses are rare but potentially fatal infections, particularly in individuals with diabetes mellitus. Management strategies vary based on the abscess size, complexity, and response to the initial treatment. We report five patients with diabetes and splenic abscesses who were managed at a tertiary care centre. All patients presented with left upper quadrant pain, fever, and leukocytosis. The diagnosis was confirmed via ultrasonography and contrast-enhanced computed tomography. One patient was managed conservatively with antibiotic therapy. Two patients underwent successful image-guided pigtail drainage, while one required splenectomy due to persistent infection. The remaining two patients underwent direct open splenectomy due to large loculated abscesses or lack of clinical improvement. The cultured isolates included *E. coli, Staphylococcus epidermidis,* and *Acinetobacter *species, and all patients recovered well. A tailored, stepwise approach to splenic abscesses, beginning with imaging, followed by medical or minimally invasive management, and escalating to surgery when necessary, yields favourable outcomes.

## Introduction

Splenic abscesses are rare, with an incidence of 0.05-0.7%, which is attributed to the spleen’s innate immunity. Early identification and aggressive treatment are essential to prevent sepsis, morbidity, and mortality, with untreated mortality rates reported as high as 70% [1,2]. Left upper quadrant pain, fever, and leukocytosis should raise suspicion [3]. Predisposing factors include immunocompromised states such as diabetes and AIDS, haematogenous spread from infective endocarditis or intravenous drug use, contiguous spread from adjacent organs, and splenic compromise such as infarction or trauma [4]. Ultrasound detects abscesses, but contrast-enhanced computed tomography (CECT) abdomen offers greater diagnostic accuracy [5]. Treatment ranges from antibiotics to splenectomy, with image-guided drainage for inaccessible unilocular abscesses. This case series reviewed five patients treated at the Sri Ramachandra Institute between March and November 2022 and aims to present the varied clinical features, diagnostic approaches, and individualised management strategies employed in patients with splenic abscess, highlighting the challenges and outcomes associated with each modality of treatment.

## Case presentation

Case 1

A 60-year-old woman with type 2 diabetes mellitus, rheumatic heart disease, and systemic hypertension presented with 21 days of left upper quadrant abdominal pain, vomiting, and a history of high-grade fever. Clinical examination revealed tachycardia and tenderness in the left hypochondrium. Laboratory investigations showed leukocytosis (20,000 cells/cumm), thrombocytosis, and a peripheral smear indicative of microcytic hypochromic anaemia with neutrophilia.

**Figure 1:**
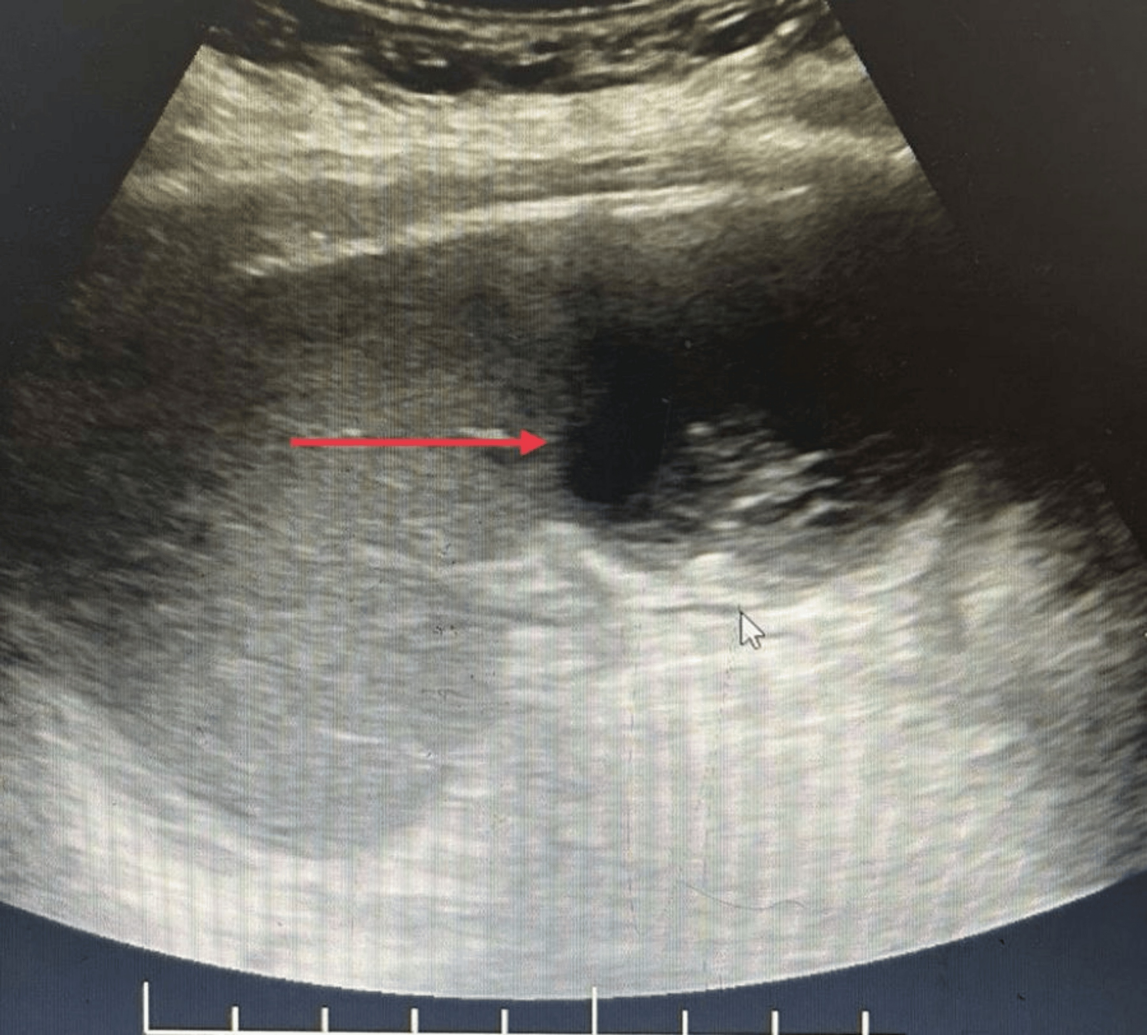
Collection in the inferior pole of the spleen (red arrow)

**Figure 2:**
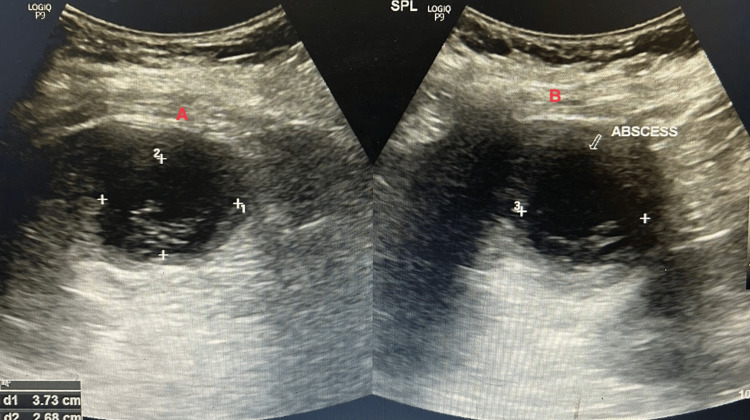
Collection of about 23 ml noted in the spleen (A), with appearance suggesting an abscess (B)

2D echocardiography showed features of rheumatic heart disease without evidence of vegetations. CECT of the abdomen revealed a 23 ml multiloculated abscess located in the inferior pole of the spleen (Figures 1, 2). Given the small size and absence of systemic compromise, the patient was managed conservatively with intravenous piperacillin-tazobactam for 10 days. She showed marked clinical improvement and was discharged after three days of hospitalisation.

The patient was followed up weekly for the first month and then monthly for three months. Follow-up included serial ultrasonography (to rule out recurrence), complete blood counts (with special attention to thrombocytosis), and monitoring for post-splenectomy complications, although no splenectomy was required in her case. The total follow-up duration was four months. No recurrence or residual collection was noted during this period.

Case 2

**Figure 3:**
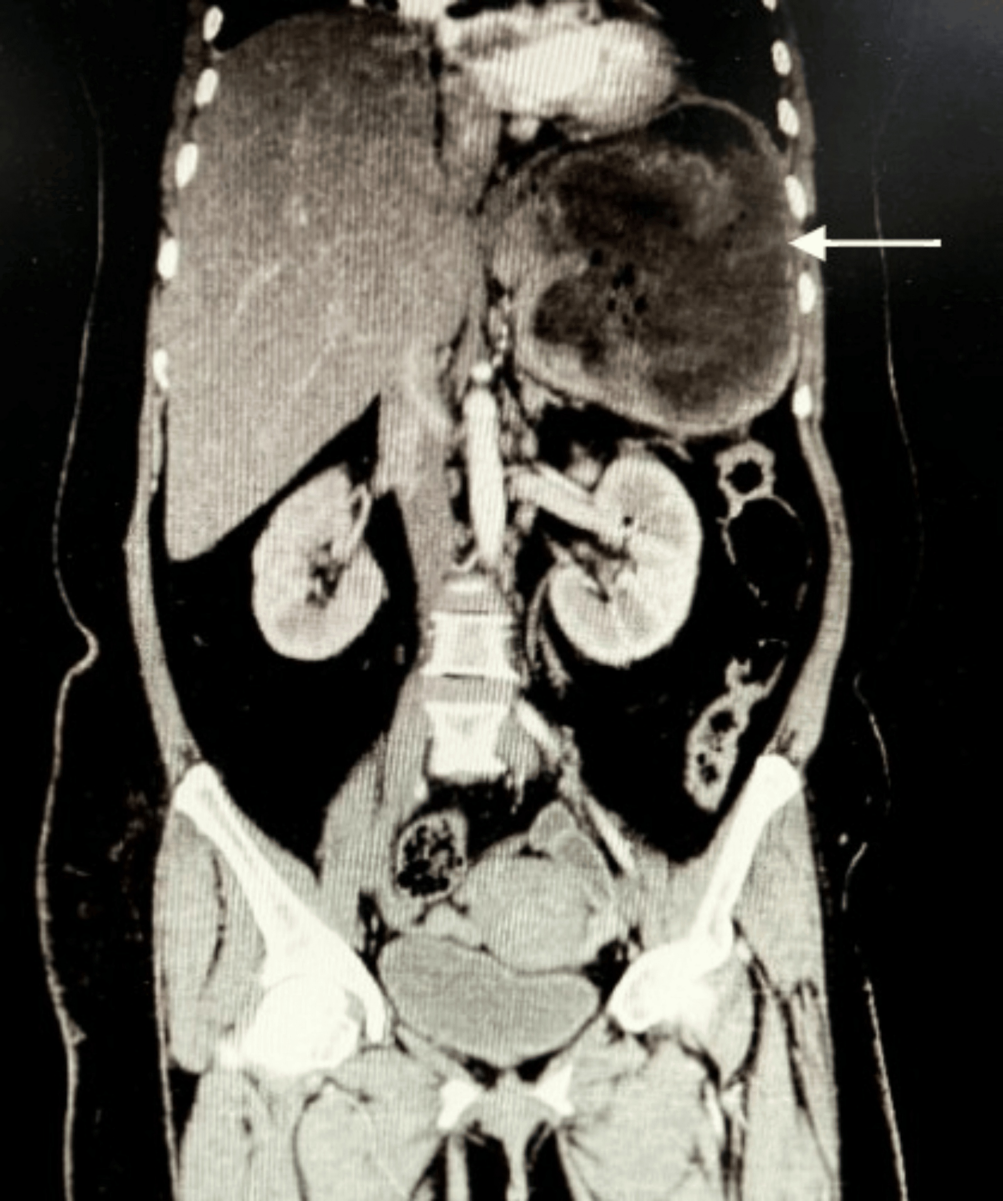
CECT whole abdomen (coronal view) showing a splenic abscess (white arrow) with air fluid levels with around 980-cc collection

**Figure 4:**
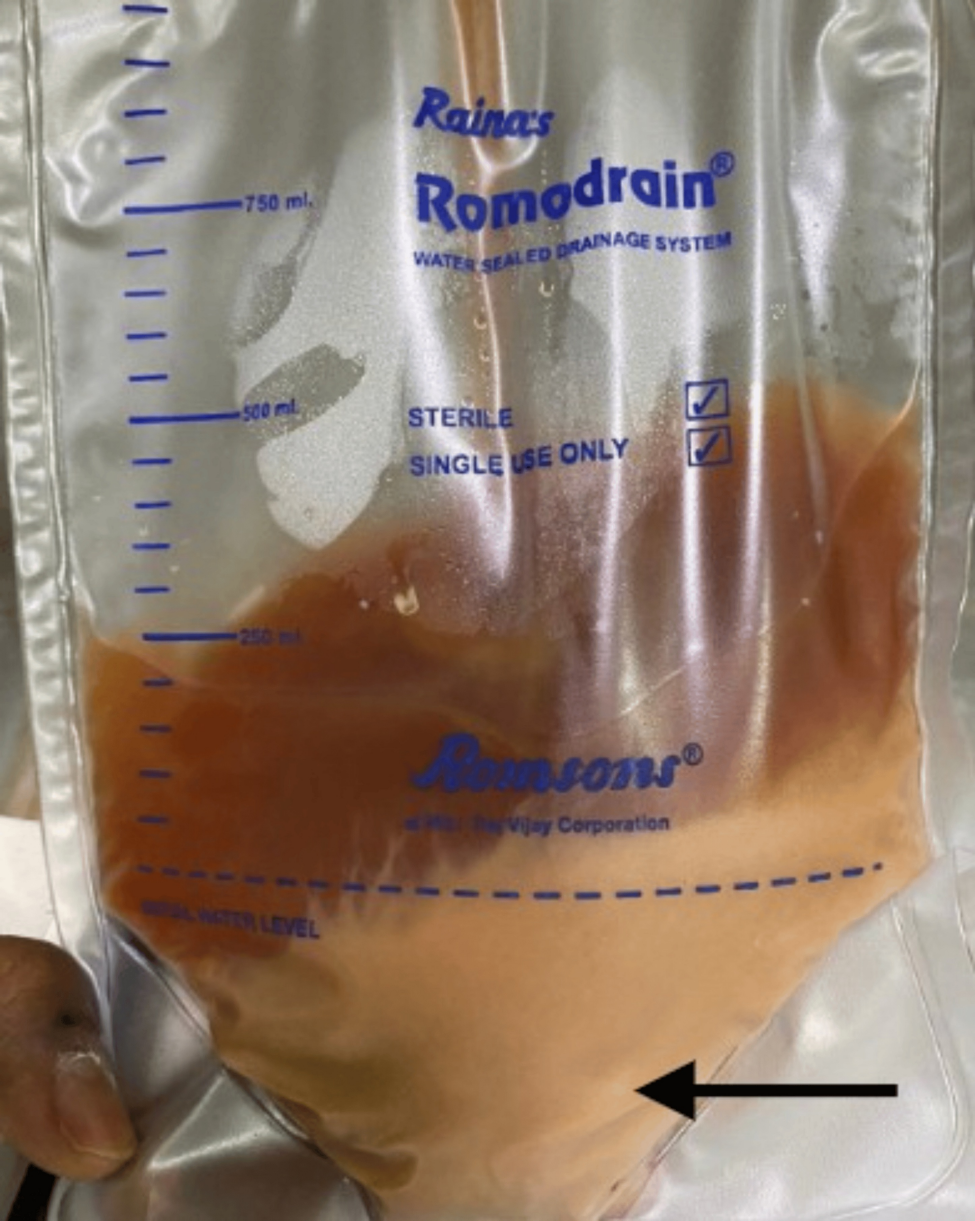
Post insertion of pigtail, the output in the drain was purulent (black arrow). Around 300 ml of purulent material is seen in the image

A 42-year-old woman with uncontrolled diabetes presented with left hypochondriac pain, fever, and loss of appetite. Investigations revealed leukocytosis (21,850 cells/cumm), thrombocytosis (974,000/µL), and microcytic hypochromic anaemia. Initial ultrasound showed a splenic abscess, and CECT revealed a 980-cc thick-walled collection with an air-fluid level and splenic vein thrombosis (Figure 3). She underwent ultrasound-guided pigtail drainage, which cultured *E. coli*, *Enterococcus faecium*, and coagulase-negative *Staphylococcus*. She received a 21-day course of piperacillin-tazobactam and linezolid, adjusted based on culture sensitivity and septic profile. Due to the recurrence of symptoms and persistence of a large collection on follow-up, a second drainage of 700 ml was performed. The catheter was removed after 11 days, and she was discharged after 35 days of anticoagulation (Figure 4).

Case 3

**Figure 5:**
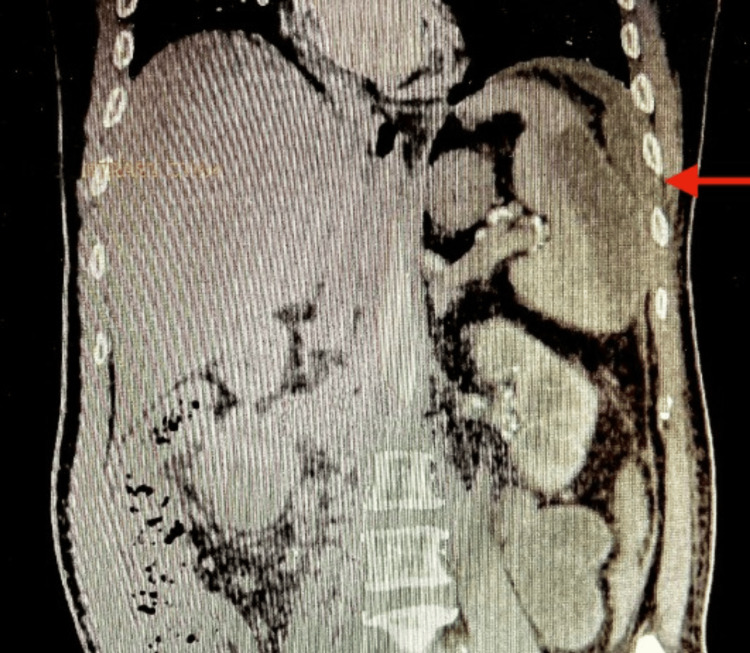
CECT whole abdomen (coronal view) shows a collection in the splenic region which is adherent to the lateral abdominal wall (red arrow)

A 57-year-old man with diabetes and a history of treated pulmonary tuberculosis presented with 10 days of abdominal pain, high-grade fever, and vomiting. Examination revealed fever, tachycardia, tachypnoea, and tenderness in the left hypochondriac and lumbar regions. Laboratory tests showed leukocytosis (21,370 cells/cu.mm). CECT revealed a 200-cc subcapsular perisplenic collection, including a 55 ml splenic parenchymal component (Figure 5). Ultrasound-guided pigtail drainage yielded 30 cc of pus, which cultured *Staphylococcus epidermidis*. Due to persistent symptoms and inadequate drainage, an open splenectomy with abscess evacuation was performed. Dense adhesions were noted intraoperatively. The patient had an uneventful postoperative recovery and received 14 days of antibiotics guided by clinical response and inflammatory markers.

Case 4

**Figure 6:**
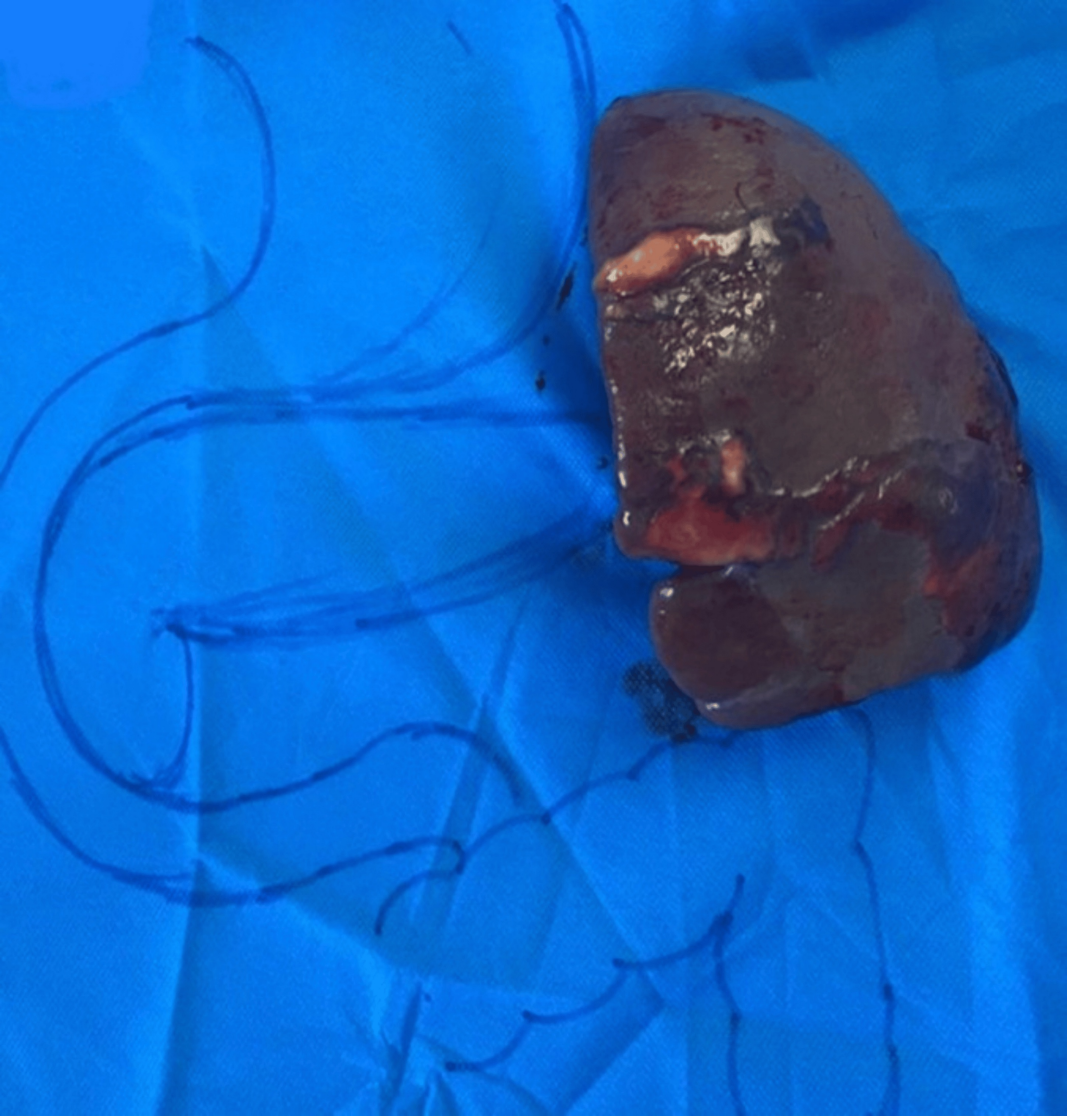
Postoperative spleen specimen of case 4

A 38-year-old man with diabetes presented with a three-month history of abdominal pain that worsened over the preceding 13 days, accompanied by fever and significant weight loss. Laboratory tests showed leukocytosis (16,930 cells/cu.mm) and microcytic hypochromic anaemia. CECT revealed a 330 ml loculated splenic abscess near the hilum. He received piperacillin-tazobactam, along with glycaemic and electrolyte correction, and was vaccinated before elective splenectomy. Intraoperatively, 100 ml of pus was drained, and the spleen was noted to be densely adherent to the transverse colon. Pus culture grew *Acinetobacter*, and histopathology confirmed an abscess. A postoperative surgical site infection was managed with antibiotics and wound care (Figure 6). The chronicity, loculated nature of the abscess, and non-response to antibiotics warranted definitive surgical intervention. He remains under regular follow-up with platelet monitoring.

Case 5

**Figure 7:**
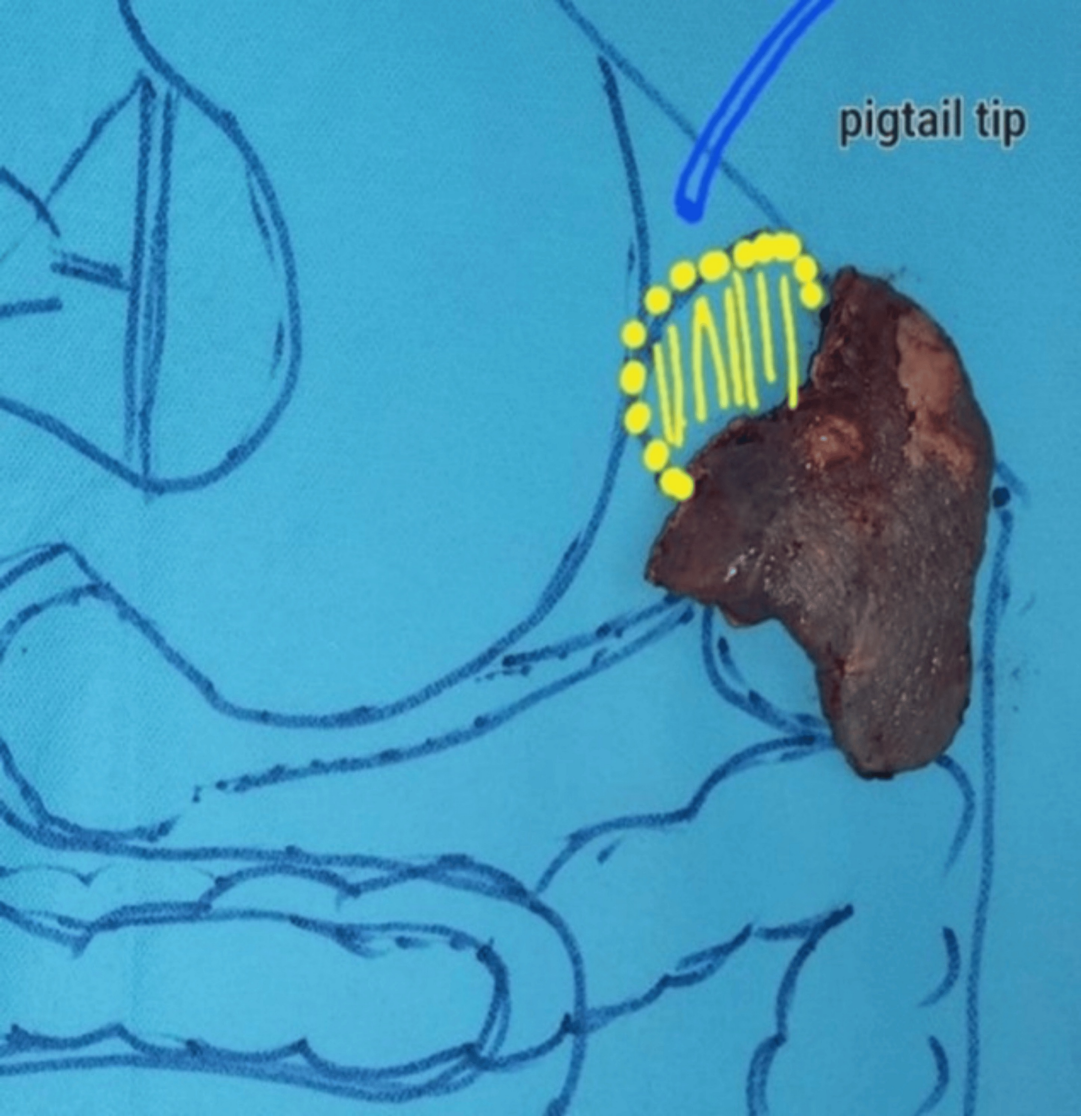
Postoperative specimen for case 5

**Figure 8:**
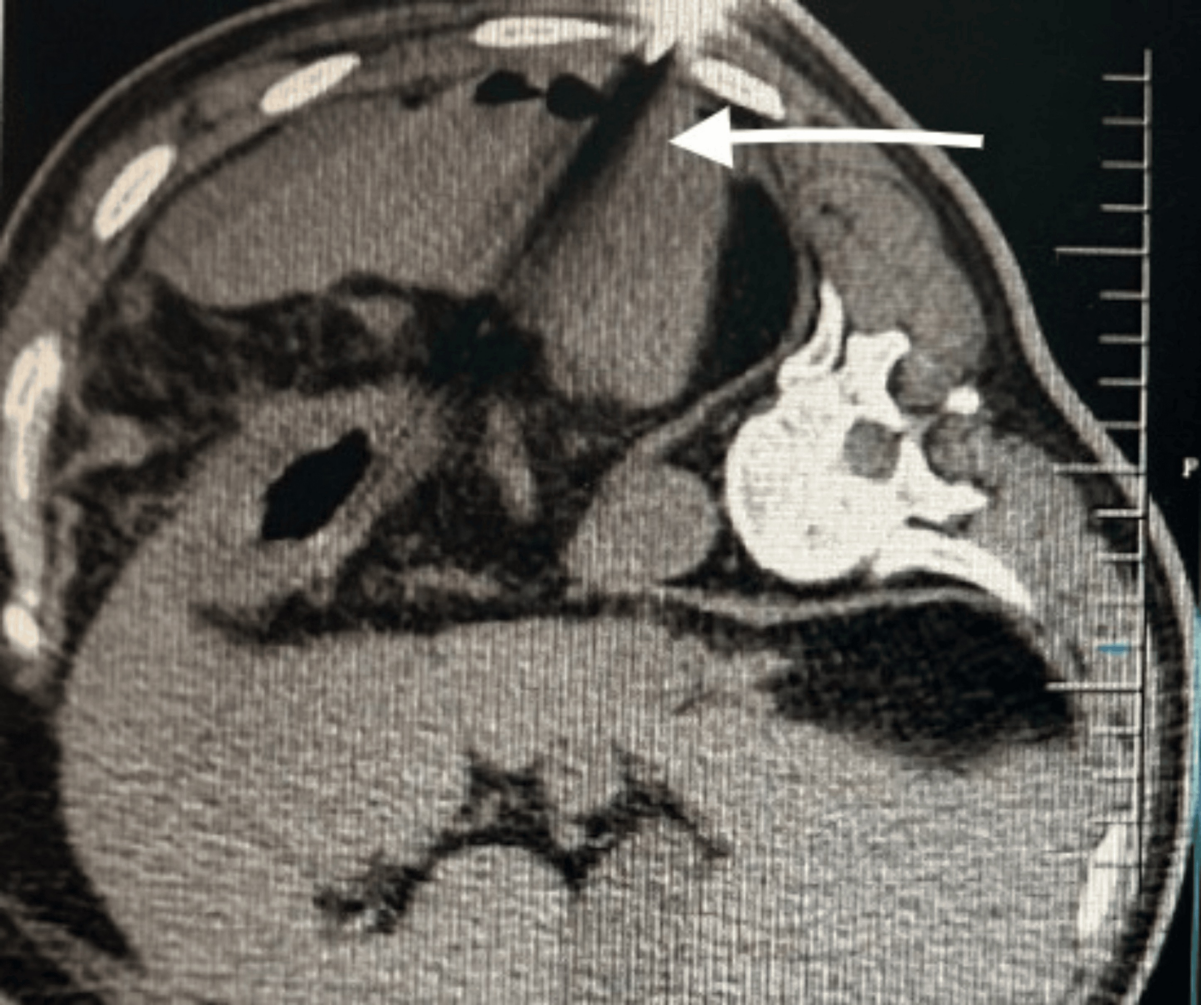
Insertion of a pigtail in a splenic abscess under CT guidance

A 70-year-old man with diabetes presented with 10 days of abdominal pain, fever, tachycardia, localised guarding in the left hypochondriac and lumbar regions, and hypotension requiring noradrenaline. Laboratory investigations showed thrombocytosis (820,000/mm³), leukocytosis (12,900/cu.mm), and microcytic hypochromic anaemia. Ultrasonography and CECT revealed multiple splenic abscesses, the largest measuring approximately 500 ml. Despite broad-spectrum antibiotics and CT-guided drainage (Figures 7, 8), the patient’s fever persisted. Due to ongoing sepsis and inadequate response, an open splenectomy was performed for definitive source control. The patient recovered well postoperatively and was transitioned to oral antibiotics following 10 days of intravenous therapy.

Follow-up

All patients were monitored post-discharge at structured intervals weekly for the initial month, followed by monthly reviews for a subsequent three-month period. The follow-up protocol included: Serial abdominal ultrasonography to detect any recurrence or residual intra-abdominal collections, complete blood count monitoring, with specific attention to post-splenectomy thrombocytosis, and evaluation for post-splenectomy infectious complications, where applicable (Table 1). The median duration of follow-up was four months, with a range of three to six months. No recurrence, persistent collections, or major complications were noted during the follow-up period.

**Table 1:** Summary of our cases 2D ECHO: Two-dimensional echocardiogram, RHD: Rheumatic Heart Disease, TB: Tuberculosis, CT: Computed Tomography, IV: Intravenous, µL: Microlitre, cumm: Cubic millimetre (mm³) All total counts and platelet counts are expressed in standard units

Parameter	Case 1	Case 2	Case 3	Case 4	Case 5
Age	60 years	42 years	57 years	38 years	70 years
Clinical features	Abdominal pain for 20 days and fever	Abdominal pain, fever, and vomiting	Abdominal pain and high-grade fever	Abdominal pain for three months, fever, and vomiting	Abdominal pain and fever for 10 days
Comorbidity	Diabetes, rheumatic disease, and systemic hypertension	Diabetes	Diabetes, pulmonary TB, and history of recurrent abscess	Diabetes	Diabetes
Total count	20,000 cells/cumm (reference range: 4,000-11,000 cells/cumm)	21,850 cells/cumm (reference range: 4,000-11,000 cells/cumm)	21,370 cells/cumm (reference range: 4,000-11,000 cells/cumm)	16,930 cells/cumm (reference range: 4,000-11,000 cells/cumm)	12,900 cells/cumm (reference range: 4,000-11,000 cells/cumm)
Platelets	2.23 × 10⁵/µL (reference range: 1.5–4.5 × 10⁵/µL)	9.74 × 10⁵/µL (reference range: 1.5–4.5 × 10⁵/µL)	2.65 × 10⁵/µL (reference range: 1.5–4.5 × 10⁵/µL)	3.33 × 10⁵/µL (reference range: 1.5–4.5 × 10⁵/µL)	8.2 × 10⁵/µL (reference range: 1.5–4.5 × 10⁵/µL)
Peripheral smear	Microcytic hypochromic anaemia with neutrophilia	Microcytic hypochromic anaemia with neutrophilic leucocytosis and thrombocytosis	Not done	Microcytic hypochromic anaemia with neutrophilic leucocytosis	Microcytic hypochromic anaemia
2D ECHO	Features of RHD	Valves were normal	Valves were normal	Valves were normal	Valves were normal
Abscess details	23 ml multiloculated abscess in the inferior pole	980 ml thick-walled collection with air-fluid level	200 ml subcapsular perisplenic collection (with 55 ml splenic parenchymal component)	330 ml loculated collection near splenic hilum	Multiple collections, largest approximately 500 ml (CT estimate)
Antibiotic treatment	10 days of IV Piperacillin–Tazobactam	21 days total (Piperacillin–Tazobactam + Linezolid), adjusted for positive blood/pus cultures and septic profile	14 days post-splenectomy, guided by response and inflammatory markers	14 days perioperatively with coverage for Acinetobacter	10 days IV antibiotics, followed by oral coverage post-splenectomy

## Discussion

Among the five patients, the median abscess volume was 200 ml. Three of the five (60%) required splenectomy due to inadequate response to conservative or interventional treatment. All patients had diabetes mellitus, and the most common presenting symptoms were left upper quadrant pain and fever. The most common symptoms among all patients were left hypochondrial pain and fever. As reported by Liu et al., a triad of fever, leucocytosis, and tenderness in the left upper quadrant of the abdomen is suggestive of splenic abscess [3]. Weight loss was also noted as a presenting complaint in some cases. All patients had diabetes, with uncontrolled diabetes recognised as a predisposing factor for splenic abscesses, as stated by Zou et al. [4]. On clinical examination, tachycardia was uniformly present, while fever was documented in most patients at initial presentation. Left hypochondrial tenderness was a consistent physical finding, reinforcing its diagnostic value in suspected splenic pathology. It is noteworthy that splenomegaly was not observed in all cases, indicating that its absence does not preclude the diagnosis of a splenic abscess.

Although ultrasound was effective in confirming the diagnosis, CT scanning more accurately quantified the collection, localised it precisely, and provided information regarding splenic vein thrombosis. Nelken et al. reported CT to have a sensitivity of 96%, superior to ultrasound’s 76% [5]. All patients exhibited elevated total leukocyte counts, with two showing thrombocytosis; 2D echocardiography ruled out infective endocarditis, and peripheral smears excluded blood dyscrasias.

Conservative management was attempted when the abscess was small. Ultrasound-guided pigtail drainage was performed in patients with uniloculated and liquefied collections at accessible sites. This less invasive approach reduces the morbidity associated with splenectomy [6]. Cultures revealed *E. coli* as the most common organism; however, Gigliotti et al. reported *Streptococcus viridians *as the most frequent organism, followed by *Klebsiella pneumoniae*. In contrast to our series, Gigliotti et al. reported *Streptococcus viridans* and *Klebsiella pneumoniae* as common isolates, whereas in our series, *E. coli* was predominant [7].

Failure to respond to conservative or minimally invasive treatments necessitated a splenectomy. Singh et al. reported favourable outcomes with this approach [8]. Liu et al. recommended that splenic abscesses larger than 4 cm be better managed with either percutaneous drainage or splenectomy [3]. In chronic inflammation with dense adhesions, open drainage and splenectomy are performed, abscess biopsy and negative fungal/AFB cultures rule out pathology, and regular follow-up monitors recurrence and thrombocytosis.

Rationale for not performing minimally invasive splenectomy

Laparoscopic splenectomy is well-established in elective settings. However, in our series, the majority of splenectomies were performed under emergent or semi-elective circumstances with findings that contraindicated a minimally invasive approach. Cases 4 and 5 demonstrated dense adhesions to adjacent viscera (transverse colon and diaphragm), making laparoscopic dissection unsafe. In Case 3, splenectomy was initially planned, but intraoperative adhesions necessitated a splenectomy and drainage instead. Thus, surgical decisions were individualised based on intraoperative findings, anatomical accessibility, and the patient’s haemodynamic stability.

Role and limitations of percutaneous catheter drainage (PCD)

PCD was attempted in two cases with varied outcomes. Case 2 required two drainage procedures but ultimately avoided splenectomy, which we considered a partial success. Case 3 experienced drainage failure and proceeded to open surgery. PCD, though less invasive, was not definitively curative in either case and should be considered a bridging or temporising measure, particularly in high-risk or unstable patients. We have tempered our earlier recommendation accordingly.

Based on our experience and current evidence, a structured approach to splenic abscess management is recommended. Conservative therapy may be appropriate for small (<3-4 cm), unilocular, and clinically silent abscesses. Early splenectomy, preferably laparoscopic when feasible, should be considered for multiloculated or large abscesses, failure of conservative or PCD, recurrent collections, or extensive splenic involvement with systemic compromise. PCD may serve as a temporising option in selected patients unfit for immediate surgery, provided close clinical monitoring is ensured.

Splenic abscesses are rare and pose diagnostic and therapeutic challenges. In the past, the standard approach consisted of intravenous antibiotics and splenectomy. Splenectomy, while definitive, carries risks of overwhelming post-splenectomy infection, especially in immunocompromised and elderly individuals. All patients in our series were diabetic, a known predisposing factor for splenic abscess. Most presented with fever, tachycardia, and left hypochondrial tenderness. While ultrasound was the initial imaging modality, CECT proved superior in quantifying abscess size, detecting splenic vein thrombosis, and guiding intervention.

In culture, *E. coli *emerged as the most common organism. Blood cultures were positive only in Case 2, growing *E. faecium* and coagulase-negative *Staphylococcus*. Pus cultures guided antibiotic therapy in all cases. Conservative therapy was successful in only one patient with a small abscess. PCD was attempted in two cases. One patient (Case 2) required two drainage procedures and prolonged antibiotics. The second (Case 3) failed drainage and required splenectomy. Thus, while PCD may serve as a temporising or selective strategy, it may not be definitive.

This case series is limited by its small sample size, retrospective nature, and absence of long-term follow-up data. The findings may not be generalisable, and further studies with larger cohorts are needed.

## Conclusions

Splenic abscesses are rare but potentially fatal, particularly in patients with diabetes mellitus. Early diagnosis using CECT is crucial to guide appropriate intervention. Management ranges from conservative therapy and image-guided drainage to splenectomy in cases that are complex or unresponsive. Post-splenectomy care, including pneumococcal vaccination and platelet monitoring, is essential to prevent long-term complications. A tailored, stepwise approach yields favourable outcomes, with treatment decisions individualised based on imaging, microbiological profile, and clinical response. In conclusion, splenic abscesses warrant an individualised management strategy. While conservative treatment and image-guided drainage may be appropriate in selected cases, early surgical intervention, preferably laparoscopic splenectomy when feasible, remains the definitive approach for larger, multiloculated, or non-resolving abscesses.
